# Relationship between Body Mass Index and Diagnosis of Overweight or Obesity in Veterans Administration Population

**DOI:** 10.3390/healthcare11111529

**Published:** 2023-05-24

**Authors:** Onur Baser, Erdem Baser, Gabriela Samayoa

**Affiliations:** 1 John D. Dingell Veterans Affairs Medical Center, Detroit, MI 48201, USA; 2 Graduate School of Public Health, City University of New York, New York, NY 10027, USA; 3 Department of Internal Medicine, University of Michigan, Ann Arbor, MI 48109, USA; 4 Columbia Data Analytics, New York, NY 10013, USA; erdem@cdanyc.com (E.B.); gabriela@cdanyc.com (G.S.)

**Keywords:** obesity, overweight, morbid obesity, veterans, underdiagnosis, BMI

## Abstract

**Background:** This paper examined the gap between obesity and its diagnosis for cohorts of patients with overweight, obesity, and morbid obesity in the Veterans Administration (VA) population. Using the risk adjustment models, it also identified factors associated with the underdiagnosis of obesity. **Methods:** Analysis was performed on a VA data set. We identified diagnosed patients and undiagnosed patients (identified through BMI but not diagnosed using ICD-10 codes). The groups’ demographics were compared using nonparametric chi-square tests. We used logistic regression analysis to predict the likelihood of the omission of diagnosis. **Results:** Of the 2,900,067 veterans with excess weight, 46% were overweight, 46% had obesity, and 8% of them had morbid obesity. The overweight patients were the most underdiagnosed (96%), followed by the obese (75%) and morbidly obese cohorts (69%). Older, male, and White patients were more likely to be undiagnosed as overweight and obese; younger males were more likely to be undiagnosed as morbidly obese. (*p* < 0.05) Comorbidities significantly contributed to diagnosis. **Conclusions**: The underdiagnosis of obesity continues to be a significant problem despite its prevalence. Diagnosing obesity accurately is necessary to provide effective management and treatment.

## 1. Introduction

By the year 2000, the human race reached a milestone, when, for the first time in history, the number of adults with excess weight surpassed those who were underweight [1,2]. The average American today is overweight or obese [3]. In the United States (US), the prevalence of obesity has been accelerating every year since the World Health Organization (WHO) declared it a pandemic in 2007, affecting individuals of all ages and all segments of society [2]. The prevalence of obesity in the US has nearly tripled in recent decades, increasing from 13% in 1960–1962 to 36.5% in 2011–2014 [4], thus affecting an estimated 60 million American adults. A 2017 report from the National Health and Nutrition Examination Survey (NHANES) showed that in the US, over 40% of young adults 20 to 39 years of age, 44% of middle-aged adults 40 to 59 years old, and 43% of older (>65 years) adults are obese [5].

Obesity is a particular concern among veterans [3]. People who have served in the US military suffer from obesity in higher numbers and overall have disproportionately poorer health status when compared with that of the older non-veteran US population. This disproportionate effect on veterans may further compound their overall risk for morbidity and mortality [3]. Of the 6 million patients who receive Veterans Administration (VA) health care yearly, 80% fall into the categories of either overweight or obese [3,6]. Previous studies done in this population have highlighted certain subgroups that are at a higher risk, such as Black women veterans, women veterans with schizophrenia, younger veterans (<65 years), and Native Hawaiian/Other Pacific Islander and American Indian/Alaska Native veterans [3,7]. The prevalence of obesity among veterans with post-traumatic stress disorder (PTSD) is higher than the prevalence of obesity among veterans overall within the VA (47% vs. 41%, respectively) [3,8].

The continued growth of the obesity epidemic is particularly concerning because obesity can have psychological, physical, and social impacts on an individual’s well-being. In addition, obesity is a significant risk factor for chronic diseases such as cardiovascular disease, hypertension, type 2 diabetes mellitus, hyperlipidemia, stroke, certain cancers, obstructive sleep apnea, liver and gallbladder disease, osteoarthritis, and gynecological problems [3,9,10,11,12,13]. Obesity and its comorbidities are a major cause of morbidity and mortality in the US [3,14]. Furthermore, it results in poor long-term health outcomes, which are costly to the affected individual, their family, and, ultimately, to the US healthcare system [3,13].

The accurate identification and diagnosis of obesity are essential for the evaluation and treatment of obesity and its associated comorbidities [15]. The US Preventive Services Task Force (USPSTF) recommends screening all adults for obesity [16]. It also recommends that once a diagnosis of obesity has been established according to body mass index (BMI) ≥ 30 kg/m^2^, the patient should be offered or referred to an interdisciplinary lifestyle intervention program [16].

Despite these recommendations and formal recognition by the American Medical Association as a disease [17], obesity continues to be underdiagnosed in clinical practice [18]. Furthermore, newly approved medications for chronic weight management have been shown in clinical trials to improve weight loss by 15% or more than 10 pounds in one year. However, since these drugs are available only by prescription, a correct diagnosis of obesity should be made. Without an appropriate prescription and insurance coverage, these drugs cost between USD 1000 and USD 1300 per month, placing a heavy economic burden on the patient. Hence, identifying undiagnosed obesity will allow more patients to access these treatments and receive the recommended care, comprising an interdisciplinary lifestyle intervention program.

This paper examines the diagnosis of overweight, obesity, and morbid obesity in VA patients and identifies factors associated with the underdiagnosis of obesity in this population. 

## 2. Methods

The VA data set used for the analysis included data from 25 million enrollees as of December 2022 and contained inpatient and outpatient files, lab information, survival, and vital statistics (e.g., height, weight, and blood pressure) collected from 152 VA hospitals, 133 VA Community Living Centers, and 958 outpatient clinics. BMI was calculated as weight (kg)/[height (m)]^2^ using the vital statistics from the data set [19]. 

A VA data set was chosen because it is one of the few which contain BMI parameters; moreover, the veteran population is at a high risk of obesity. BMI (weight in kilograms divided by the square of height in meters [kg/m^2^]) is a simple, low-cost indirect measure for assessing obesity with reasonable height standardization. The BMI cutoffs defining obesity are based on well-established risks for cardiometabolic morbidity and premature mortality [20].

Three groups of patients were defined using the BMI: overweight (25 kg/m^2^ to <30 kg/m^2^), obese (30 kg/m^2^ to <40 kg/m^2^), and morbidly obese (≥40 kg/m^2^). Among these patients, diagnosed patients were classified according to ICD-10 codes as follows:

- Overweight identified using E66.3, Z68.25, Z68.26, Z68.27, Z68.28, Z68.29, and Z68.
- Obesity identified using E66.9, E66.09, E66.1, E66.8, Z68.3, and Z68.54
- Morbid obesity identified using E66.01, E66.2, Z68.4, and Z68.54

We then identified two cohorts: Diagnosed Patients and Undiagnosed Patients (patient groups identified through BMI but not diagnosed using ICD-10 codes). The two groups were compared based on demographics (sex, age, race, and comorbidities) using nonparametric chi-square tests. We used logistic regression analysis to predict the likelihood of the omission of diagnosis of all obese patients. Age, gender, race, and comorbidities such as coronary artery disease, hypertension, hyperlipidemia, diabetes, sleep apnea, osteoarthritis, hyperuricemia, gallbladder disease, and mental disorders were used as explanatory variables, and each variable’s impact on the odds ratio (OR) of the omission of the diagnosis was calculated. An alpha level of 0.05 was used as the threshold level of significance. All statistical analyses were performed using SAS 9.4 (SAS Institute, Cary, NC, USA).

## 3. Results

A total of 2,900,067 veterans had excess weight problems: 46% of these veterans were overweight, 46% had obesity, and 8% of them had morbid obesity. The overweight population was the most underdiagnosed based on ICD-10 codes. Of 1,333,473 patients who were overweight, only 57,675 (4%) were diagnosed with ICD-10 codes as overweight at VA facilities. Of 1,343,968 obese patients, only 329,338 patients (25%) were diagnosed as obese. Of the total 222,626 patients with morbid obesity, only 63,380 patients (31%) were diagnosed as having morbid obesity (Table 1). 

For the overweight groups, relative to diagnosed patients, undiagnosed patients were more likely to be older (63.98 years vs. 61.11 years, *p* < 0.001), male (93.54% vs. 88.98%, *p* < 0.001), and White (71.36% vs. 69.24%, *p* < 0.001). Additionally, undiagnosed patients were more likely to have hypertension (40.86% vs. 43.25%, *p* < 0.001), hyperlipidemia (29.09% vs. 34.67%, *p* < 0.001), diabetes (20.71% vs. 24.04%, *p* < 0.001), sleep apnea (6.92% vs. 10.68%, *p* < 0.001), osteoarthritis (11.46% vs. 12.83%, *p* < 0.001), hyperuricemia (0.84% vs. 1.21%, *p* < 0.001), and mental disorders (37.21% vs. 42.16%, *p* < 0.001). There were no statistical differences in coronary artery disease (7.12% vs. 7.01%, *p* = 0.2731) and gallbladder disease (0.06% vs. 0.08%, *p* = 0.0952) (Table 2). Among overweight patients diagnosed with BMI, 104,114 had no comorbidities; by contrast, 4187 undiagnosed overweight patients had no comorbidities. 

Results for the obese groups were similar to the overweight groups except for comorbidities. For the obese groups, relative to diagnosed patients, undiagnosed patients were more likely to be older (61.63 years vs. 59.45 years, *p* < 0.001), male (92.69% vs. 89.64%, *p* < 0.001), White (70.86% vs. 68.80%, *p* < 0.001), and more likely to have coronary artery disease (7.06% vs. 7.52%, *p* < 0.0001), hypertension (45.91% vs. 50.33%, *p* < 0.001), hyperlipidemia (30.95% vs. 36.26%, *p* < 0.001), diabetes (28.83% vs. 33.71%, *p* < 0.001), sleep apnea (14.02% vs. 21.97%, *p* < 0.001), osteoarthritis (13.57% vs. 16.00%, *p* < 0.001), hyperuricemia (1.02% vs. 1.49%, *p* < 0.001), and mental disorders (38.01% vs. 42.73%, *p* < 0.001). There were no statistical differences in gallbladder disease (0.06% vs. 0.08%, *p* = 0.0008). No comorbidities were present in the 78,146 patients diagnosed with obesity based on BMI, while the 21,811 undiagnosed patients with obesity had no comorbidities. 

For morbidly obese patients, unlike the obese and overweight group, relative to the diagnosed group, undiagnosed patients were slightly younger (59.00 years vs. 59.22 years, *p* < 0.0001) and were more likely to be male (88.68% vs. 90.32%, *p* < 0.001) and White (68.73% vs. 70.78%, *p* < 0.001). Having a comorbidity increased the likelihood of diagnosis. These patients were more likely to have coronary artery disease (6.85% vs. 9.19%, *p* < 0.0001), hypertension (52.70% vs. 61.85%, *p* < 0.001), hyperlipidemia (32.31% vs. 39.01%, *p* < 0.001), diabetes (39.55% vs. 49.03%, *p* < 0.001), sleep apnea (28.56% vs. 42.21%, *p* < 0.001), osteoarthritis (16.62% vs. 22.57%, *p* < 0.001), hyperuricemia (1.25% vs. 1.78%, *p* < 0.001), and mental disorders (40.40% vs. 46.67%, *p* < 0.001). There were no statistical differences in gallbladder disease (0.06% vs. 0.09%, *p* = 0.0095). Of the patients diagnosed with morbid obesity based on BMI, 9969 had no comorbidities compared with 3218 undiagnosed patients with morbid obesity with no comorbidities. 

Results of logistic regression analyses indicate that the patient characteristics assessed were predictive of an obesity diagnosis (including overweight, obesity, or morbid obesity). As the ORs indicated, overweight, obese, and morbidly obese patients were more likely to be undiagnosed if they were older (OR = 1.31, 1.44, and 1.14, respectively) and male (OR = 1.65, 1.40, and 0.92, respectively). In the obese population, patients were more likely to be undiagnosed if they were White (OR = 0.95; 95% confidence interval [CI}, 0.93–0.97). Among races other than White or Black, patients with morbid obesity were more likely to be undiagnosed (OR= 0.94; 95% CI, 0.88–0.99) (Table 3).

Any indicated comorbidity increased the likelihood of an obesity diagnosis, including coronary artery disease and gallbladder disease. However, in our sample, we still found some patients without comorbidities, but they were a minority. Therefore, comorbidity consistently increased the likelihood of diagnosis across the groups after controlling for age, gender, and race. As a result, the majority of the ORs are less than 1 and are significant. Patients in the overweight and obese population were more likely to be diagnosed if they had hypertension (OR = 0.97 [95% CI, 0.95–0.99]; OR= 0.92 [95% CI, 0.91–0.93], respectively). However, for the population with morbid obesity, patients were more likely to be diagnosed if they had hyperlipidemia (OR = 0.94 [95% CI, 0.93–0.97]). Patients in the overweight, obese, and morbidly obese groups were all more likely to be diagnosed if they had a mental disorder (OR= 0.94 [95% CI, 0.93–0.96]; OR= 0.97 [95% CI, 0.96–0.98], OR= 0.90 [95% CI, 0.89–0.92], respectively).

## 4. Discussion

It is estimated that less than 30% of adults with obesity receive a diagnosis during their primary care physician visits [18]. The present study confirmed that obesity is clearly underdiagnosed in both genders and across all age groups and races within the VA population. Our data showed that 69% of morbidly obese patients were undiagnosed, 75% of obese patients were underdiagnosed, and nearly all the overweight patients (96%) went undiagnosed. Our findings are consistent with those of Betancourt et al. [3], who examined the obesity and morbidity risk in the US veteran population and found that 69.7% of the population had obesity/overweight status, similar to the 69% of undiagnosed morbidly obese patients in our study. This highlights that patients’ overweight or obese status according to BMI does not necessarily translate to clinical practice and administrative data, therefore increasing the likelihood of not receiving proper treatment. However, comparisons must be interpreted with the understanding that VA data reflect a treatment-seeking population, who may be older and sicker than the general population [7].

Several reasons have been attributed to the underdiagnosis of obesity, including healthcare providers’ (HCPs’) perception that obesity is not a disease; low expectations for patient success; lack of time or knowledge to provide appropriate advice regarding nutrition, societal stigma, or concerns with denials of payment for services; and limited therapeutic tools to treat patients with obesity [21]. A recent survey of people with obesity (*n* = 3008) and HCPs (*n* = 606) discovered that although obesity is perceived as a disease by the majority of people with obesity (65%) and HCPs (80%), providers and patients struggle with talking about weight for several reasons [15,22]. Patients report that they do not seek help from their HCP because they believe it is their personal responsibility (44%), they already know what to do (37%), and/or they do not have financial means to support a weight loss effort (23%) [15,22]. The primary reasons HCPs do not initiate discussions about weight loss are time constraints (52%) and having more critical issues to discuss with the patient (45%) [15,22]. Other HCPs cite that the patient was not motivated to lose weight (27%) or was not interested in weight loss (26%), and/or expressed concern about the patient’s emotional state or psychological issues (22%) [15,22]. Furthermore, recognition and coding for obesity in civilian healthcare settings are poor [15]. Previous studies have shown that among patients who met the objective criteria for obesity, few were diagnosed with an obesity code [15,23,24] or their clinical visits lacked a complete height and weight record to facilitate calculating BMI [15]. Additionally, another study showed that patients with known comorbidities (e.g., type 2 diabetes, hypertension, sleep apnea) were not diagnosed with obesity [15,22]. Additionally, some studies indicated that older patients and men were significantly less likely to have an obesity diagnosis, and the presence of an obesity diagnosis was the strongest predictor of creating a plan to address obesity [15,25].

Our data add to the literature indicating that the underdiagnosis of obesity could be attributable to the low documentation of overweight and obesity due to under-coding and the general perception that these conditions are not identified as a significant problem in primary care [15,23]. Similarly, Mattar et al. [22] examined the prevalence of obesity documentation among patients with corresponding BMI as contained in patients’ electronic medical records and found that 5.6% of patient records listed obesity as a problem.

Furthermore, a reason why morbid obesity is more likely to be diagnosed could be the visual undeniability of the problem’s severity [23]. Additionally, these results indicate that providers may often wait until the health condition becomes severe enough to warrant an ICD-10 diagnosis.

There was a clear demographic divide among our overweight, obese, and morbidly obese patient population as to whether they received a diagnosis. Older, male, and White patients were more likely to have undiagnosed overweight and obesity, while younger male patients were more likely to have undiagnosed morbid obesity. This finding is consistent with a recent study of nearly 5 million VA primary care patients (347,112 females; 4,567,096 males), of whom 37% were overweight and 41% were obese [7,9]. In that study, obesity was common among male veterans 18 to 44 years of age [7]. Our data support these statistics, showing that patients with obesity and morbid obesity were more likely to be male (295,498 males and 61,763 males, respectively). However, in contrast with that study, our statistics found that older patients (>65 years) were undiagnosed with obesity or morbid obesity (147,232 males and 27,934 males, respectively). Again, this discrepancy could be due to VA data reflecting a treatment-seeking population that may be older and sicker than the general population [7].

Additionally, the discrepancies involving gender and age could be explained by weight-related beliefs and behaviors. The younger male veterans may have the perception that men are heavier due to muscle mass, although with a BMI of 30 or higher, the correlation with obesity is strong [15].

It is important to note that our study analyzed the relationship between BMI and diagnosis of overweight or obesity in a VA population. However, other obesity measurements have been devised in recent years, such as waist circumference, waist-to-hip ratio, waist-to-height ratio, abdominal volume index, conicity index, mid-arm muscular area, and visceral adiposity index [26,27]. Some of these indices have been shown to be good determinators for cardiovascular risk and metabolic syndrome [26]. The close relationship between obesity and cardiometabolic health is a well-known issue, and central obesity represents the cornerstone of a metabolic syndrome diagnosis. Both obesity and metabolic syndrome are associated with an increased risk of cardiovascular disease and type 2 diabetes [26,27]. These simple anthropometric and central obesity indices are still being studied to determine their reliability and the clinical validation of these results despite the high predictive value of these discriminators [26]. Therefore, future research is warranted when the data indicates whether the predictive power of different indices to diagnose obesity varies.

Moreover, our risk adjustment models captured the differences in age, gender ethnicity, and health status via comorbidities. Other factors may affect obesity, such as economic status, marital status, education preference, or residential area classification; however, our data set did not include these variables. Therefore, is important to note that to the extent that our current explanatory variable set does not capture the other socioeconomic factors, our estimates may be biased.

Our study highlights that comorbidities can signify a diagnosis of obesity or morbid obesity. Hypertension, hyperlipidemia, diabetes, mental disorders, and osteoarthritis were significantly associated with a diagnosis of overweight, obesity, and morbid obesity. Furthermore, a multivariate analysis study [23] showed that in addition to age and gender, morbid obesity and the cumulative number of comorbidities were significantly associated with obesity documentation. Nonetheless, this highlights the concern that delaying the proper diagnosis of obesity until a comorbidity is diagnosed might delay prevention, health education, and early weight management and treatment.

It is important to note that the negative connotation of being labeled “obese” might imply an adverse effect on a population that is susceptible to depression [3,9]. Our findings support data showing that mental disorders are a predominant comorbidity in the veteran population; among all the groups, overweight, obese, and morbidly obese patients were more likely to be diagnosed if they had a mental disorder (OR = 0.94 [95% CI, 0.93–0.96]; OR = 0.97 [95% CI, 0.96–0.98], OR = 0.90 [95% CI, 0.89–0.92], respectively). The fact that many veteran patients might have a mental disorder could make the discussion of obesity a sensitive and potentially difficult topic for an HCP to discuss with their patients, thereby increasing the likelihood of underdiagnosis. It is important to highlight that the time constraints of patient care and the prioritization of discussing matters such as cardiovascular disease over obesity per se may be a complicated factor. Further studies of these factors are needed to help address the causes of underdiagnosis of obesity. Regardless, obesity should be identified and addressed early to prevent morbidity and mortality from associated medical conditions.

In an effort to stem the tide of the obesity epidemic, the VA healthcare system offers various weight management programs [3,9]. Some VA initiatives aimed at providing veterans the tools to better manage their weight include education on proper nutrition and the benefit of regular exercise, the use of technology such as daily apps, medications, and, when warranted, bariatric surgery [3]. However, these interventions are not being properly utilized due to the underdiagnosis of obesity in the veteran population.

## 5. Limitations

This study has several limitations related to the use of administrative data sets, which may be subject to inaccurate coding of patient clinical diagnoses and procedures, with clinical information limited to conditions and treatments defined by ICD-10-CM codes. In addition, some limitations, as previously discussed, are that our data set did not contain information about marital status, education level, or residential area classification, which might play an important role in obesity. Our data did capture differences in age, gender, race, and comorbidities, as discussed above.

Furthermore, a consensus is still lacking among experts regarding how to define and measure obesity properly. While BMI is the accepted standard, it has been shown to have various limitations. First, BMI is an indirect measure of body fat and has been shown to have high specificity but low sensitivity to identify adiposity [28]. In addition, BMI measurements do not factor in age-related changes in body composition such as increased body fat and decreased muscle mass [29].

We have studied the VA population. Although the data set is nationally representative, it is predominantly male and comprises a vulnerable population. Replication of this study using other data sets that are more representative in terms of gender and income distribution would be useful.

## 6. Conclusions

Despite obesity’s high prevalence in the US veteran population, underdiagnosis continues to be a significant problem. Additionally, our study findings highlight how obesity is improperly coded, which could be a reason for low documentation. Furthermore, we identified predictors of obesity documentation such as age, gender, and comorbidities. Specifically, we demonstrated that obesity is underdiagnosed, but those with morbid obesity were much more likely to be diagnosed than overweight patients. This finding could be associated with the visual undeniability of the problem severity and the fact that morbidly obese patients tend to have more severe associated comorbidities. Nonetheless, obesity is a modifiable risk factor for multiple comorbidities that, when promptly and properly treated, can improve the overall health of the patient, and lower healthcare costs. Therefore, it is crucial to diagnose obesity accurately to provide effective management, improving patients’ overall quality of life. Additionally, it is important to address the factors associated with underdiagnosis of obesity. For example, BMI as a core measure of vital signs is not fully harnessed and applied in the delivery of health care. Another factor contributing to the underdiagnosis of obesity could be the low documentation of obesity in the administrative data and electronic health records, as shown in our data.

Even though BMI measurement cannot be a reliable measurement of obesity in some populations, it is still a simple, low-cost indirect measure for assessing obesity with reasonable height standardization. Additionally, the BMI cutoffs defining obesity are based on well-established risks for cardiometabolic morbidity and premature mortality [20]. Moreover, BMI is the metric currently in use for defining anthropometric height/weight characteristics in adults and for classifying (categorizing) them into groups [30]. In addition, it is widely used in determining public health policies [30]. The BMI has been useful in population-based studies by virtue of its wide acceptance since the World Health Organization (WHO) classification of body weight for height, based on the BMI, was published in 1995 in defining specific categories of body mass as a health issue [30].

Further studies are recommended to investigate the underlying causes of lower rates of obesity documentation for certain sociodemographic groups. Moreover, studies looking at physician-specific factors that play a role in determining diagnosed obesity are warranted. Especially with the availability of new treatments, insurance companies require a diagnosis for coverage. Therefore, closing the underdiagnosis gap is important.

## Figures and Tables

**Table 1:** Total Veteran Population with Overweight, Obesity, and Morbid Obesity.

	Overweight (BMI > 25 to 29	Obesity (BMI ≥ 30 to 39)	Morbid Obesity (BMI ≥ 40)
**BMI measurement**	1,274,692	1,014,330	154,246
**ICD-10 codes**	58,781	329,638	68,380
**Total population**	1,333,473	1,343,968	222,626

**Table 2:** Characteristics of the Veterans’ Population.

	**Overweight by BMI (N = 1,274,692)**		**Overweight by ICD 10 Code ** **(N = 58,781)**		***p*** **Value**	**Obese by BMI ** **(N = 1,014,330)**		**Obese by ICD 10 Code ** **(N = 329,638)**		***p*** **Value**	**Morbidly Obese by BMI (N = 154,246)**		**Morbidly Obese by ICD 10 Code ** **(N = 68,380)**		***p*** **Value**
	**N/Mean**	**%/Std**	**N/Mean**	**%/Std**		**N/Mean**	**%/Std**	**N/Mean**	**%/Std**		**N/Mean**	**%/Std**	**N/Mean**	**%/Std**	
Age (years)	63.98	15.71	61.22	14.58	<0.0001	61.63	14.61	59.45	13.64	<0.0001	59.00	13.19	59.22	11.90	0.0001
18–45	182,464	14.31%	9284	15.79%	<0.0001	157,969	15.57%	56,166	17.04%	<0.0001	26,084	16.91%	9588	14.02%	<0.0001
46–54	117,419	9.21%	7154	12.17%	<0.0001	124,346	12.26%	49,427	14.99%	<0.0001	25,027	16.23%	11,562	16.91%	0.0001
55–64	231,203	18.14%	12,692	21.59%	<0.0001	204,899	20.20%	76,813	23.30%	<0.0001	37,871	24.55%	19,296	28.22%	<0.0001
65+	743,606	58.34%	29,651	50.44%	<0.0001	527,116	51.97%	147,232	44.66%	<0.0001	65,264	42.31%	27,934	40.85%	<0.0001
Gender															
Male	1,192,292	93.54%	52,305	88.98%	<0.0001	940,225	92.69%	295,498	89.64%	<0.0001	136,790	88.68%	61,763	90.32%	<0.0001
Female	82,400	6.46%	6476	11.02%	<0.0001	74,105	7.31%	34,140	10.36%	<0.0001	17,456	11.32%	6617	9.68%	<0.0001
Race															
White	909,577	71.36%	40,698	69.24%	<0.0001	718,767	70.86%	226,780	68.80%	<0.0001	106,017	68.73%	48,398	70.78%	<0.0001
Black	202,654	15.90%	10,787	18.35%	<0.0001	173,197	17.08%	65,461	19.86%	<0.0001	30,538	19.80%	13,296	19.44%	0.0527
Other	41,125	3.23%	2152	3.66%	<0.0001	30,437	3.00%	9895	3.00%	0.9749	4828	3.13%	1839	2.69%	<0.0001
Unknown	121,336	9.52%	5144	8.75%	<0.0001	91,929	9.06%	27,502	8.34%	<0.0001	12,863	8.34%	4847	7.09%	<0.0001
Comorbidities															
Coronary artery disease	90,816	7.12%	4118	7.01%	0.2731	71,596	7.06%	24,801	7.52%	<0.0001	10,568	6.85%	6286	9.19%	<0.0001
Hypertension	520,865	40.86%	25,425	43.25%	<0.0001	465,660	45.91%	165,903	50.33%	<0.0001	81,292	52.70%	42,291	61.85%	<0.0001
Hyperlipidemia	370,845	29.09%	20,380	34.67%	<0.0001	313,911	30.95%	119,540	36.26%	<0.0001	49,843	32.31%	26,675	39.01%	<0.0001
Diabetes	263,984	20.71%	14,133	24.04%	<0.0001	292,407	28.83%	111,105	33.71%	<0.0001	61,012	39.55%	33,530	49.03%	<0.0001
Sleep apnea	88,179	6.92%	6279	10.68%	<0.0001	142,258	14.02%	72,405	21.97%	<0.0001	44,050	28.56%	28,862	42.21%	<0.0001
Osteoarthritis	146,090	11.46%	7540	12.83%	<0.0001	137,617	13.57%	52,730	16.00%	<0.0001	25,637	16.62%	15,433	22.57%	<0.0001
Hyperuricemia	10,649	0.84%	714	1.21%	<0.0001	10,306	1.02%	4901	1.49%	<0.0001	1932	1.25%	1220	1.78%	<0.0001
Gallbladder disease	756	0.06%	45	0.08%	0.0952	595	0.06%	249	0.08%	0.0008	88	0.06%	60	0.09%	0.0095
Mental disorders	474,257	37.21%	24,783	42.16%	<0.0001	385,534	38.01%	140,849	42.73%	<0.0001	62,311	40.40%	31,914	46.67%	<0.0001
None	104,114	8.17%	4187	7.12%	<0.0001	78,146	7.70%	21,811	6.62%	<0.0001	9969	6.46%	3218	4.71%	<0.0001

**Table 3:** Odds Ratios of Clinical Features of Undiagnosed Obesity by Severity of Obesity.

	Overweight				Obese				Morbidly Obese			
	Odds Ratio	Z-Value	95% Confidence Limits		Odds Ratio	Z-Value	95% Confidence Limits		Odds Ratio	Z-Value	95% Confidence Limits	
			Lower	Upper			Lower	Upper			Lower	Upper
Age (years)												
46–54	0.8914	0.000	0.8632	0.9206	0.9908	0.212	0.9765	1.0053	0.9269	0.000	0.8967	0.9581
55–64	0.9973	0.856	0.9690	1.0265	1.0918	0.000	1.0772	1.1066	0.9196	0.000	0.8914	0.9487
65+	1.3138	0.000	1.2787	1.3500	1.4359	0.000	1.4177	1.4544	1.1389	0.000	1.1042	1.1747
Gender												
Male	1.6534	0.000	1.6073	1.7008	1.4002	0.000	1.3806	1.4202	0.9211	0.000	0.8927	0.9504
Race												
White	1.0656	0.005	1.0189	1.1144	0.9505	0.000	0.9285	0.9731	0.8311	0.000	0.7861	0.8787
Black	1.0355	0.152	0.9873	1.0862	0.9091	0.000	0.8869	0.9318	0.9096	0.001	0.8581	0.9643
Unknown	1.1188	0.000	1.0624	1.1783	0.9904	0.480	0.9644	1.0172	0.9375	0.048	0.8794	0.9994
Comorbidities												
Coronary artery disease	1.0775	0.000	1.0417	1.1146	1.0126	0.120	0.9967	1.0287	0.9015	0.000	0.8710	0.9330
Hypertension	0.9692	0.002	0.9500	0.9889	0.9166	0.000	0.9078	0.9254	0.8708	0.000	0.8517	0.8903
Hyperlipidemia	0.7763	0.000	0.7612	0.7917	0.8484	0.000	0.8405	0.8564	0.9480	0.000	0.9282	0.9682
Diabetes	0.8312	0.000	0.8136	0.8491	0.8099	0.000	0.8023	0.8177	0.7784	0.000	0.7622	0.7949
Sleep apnea	0.6763	0.000	0.6578	0.6954	0.6252	0.000	0.6187	0.6317	0.6231	0.000	0.6108	0.6357
Osteoarthritis	0.9379	0.000	0.9144	0.9621	0.8951	0.000	0.8850	0.9053	0.7834	0.000	0.7653	0.8019
Hyperuricemia	0.7711	0.000	0.7142	0.8326	0.7760	0.000	0.7495	0.8035	0.8402	0.000	0.7806	0.9043
Gallbladder disease	0.8745	0.384	0.6467	1.1826	0.8631	0.054	0.7430	1.0026	0.7685	0.121	0.5507	1.0724
Mental disorders	0.9410	0.000	0.9242	0.9581	0.9729	0.000	0.9646	0.9813	0.9028	0.000	0.8854	0.9205
None	0.9662	0.049	0.9336	0.9998	0.9156	0.000	0.9004	0.9310	0.8515	0.000	0.8149	0.8899

## Data Availability

Data are not available due to privacy and ethical restrictions.
